# Investigation of the Role of Dinutuximab Beta-Based Immunotherapy in the SIOPEN High-Risk Neuroblastoma 1 Trial (HR-NBL1)

**DOI:** 10.3390/cancers12020309

**Published:** 2020-01-28

**Authors:** Ruth Ladenstein, Ulrike Pötschger, Dominique Valteau-Couanet, Roberto Luksch, Victoria Castel, Shifra Ash, Geneviève Laureys, Penelope Brock, Jean Marie Michon, Cormac Owens, Toby Trahair, Godfrey Chi Fung Chan, Ellen Ruud, Henrik Schroeder, Maja Beck-Popovic, Guenter Schreier, Hans Loibner, Peter Ambros, Keith Holmes, Maria Rita Castellani, Mark N. Gaze, Alberto Garaventa, Andrew D.J. Pearson, Holger N. Lode

**Affiliations:** 1 St. Anna Children‘s Hospital and Children’s Cancer Research Institute (CCRI), Department of Paediatrics, Medical University, 1090 Vienna, Austria; 2 Children’s Cancer Research Institute (CCRI), St. Anna Kinderkrebsforschung, Department of Paediatrics, Medical University, 1090 Vienna, Austria; ulrike.poetschger@ccri.at (U.P.); peter.ambros@ccri.at (P.A.); 3 Children and Adolescent Oncology Department, Gustave Roussy, Paris-Sud, University, 94805 Villejuif, France; Dominique.VALTEAU@gustaveroussy.fr; 4 Fondazione IRCCS Istituto Nazionale dei Tumori, 20133 Milan, Italy; roberto.luksch@istitutotumori.mi.it (R.L.); rita.castellani@istitutotumori.mi.it (M.R.C.); 5 Hospital Universitario y Politecnico La Fe, 46026 Valencia, Spain; castel_vic@gva.es; 6 Schneider Children‘s Medical Center of Israel, Sackler Faculty of Medicine Tel Aviv University, Petach, Tikvah 49202, Israel; shifraa@clalit.org.il; 7 University Hospital Ghent, 9000 Ghent, Belgium; genevieve.laureys@ugent.be; 8 Great Ormond Street Hospitalfor Children, London WC1N 3JH, UK; peppybrock@gmail.com; 9 Institut Curie, 75248 Paris, France; jean.michon@curie.fr; 10 Paediatric Haematology/Oncology, Our Lady’s Children’s Hospital, Crumlin, D12 N512 Dublin, Ireland; cormac.owens@olchc.ie; 11 Sydney Children’s Hospital, Randwick NSW 2031, Australia; Toby.Trahair@SESIAHS.HEALTH.NSW.GOV.AU; 12 Department of Paediatrics & Adolescent Medicine, Queen Mary Hospital, Hong Kong, China; gcfchan@hku.hk; 13 Rikshospitalet, 0027 Oslo, Norway; ellen.ruud@rikshospitalet.no; 14 Department of Paediatrics, University Hospital of Aarhus, 8200 Aarhus, Denmark; henrik.schroeder@skejby.rm.dk; 15 Department of Paediatrics, University Hospital Lausanne, 1011 Lausanne, Switzerland; Maja.Beck-Popovic@chuv.ch; 16 AIT Austrian Institute of Technology GmbH, 8020 Graz, Austria; guenter.schreier@ait.ac.at; 17 Apeiron Biologics AG, 1030 Vienna, Austria; hans.loibner@hl-bioscience.com; 18 St George’s Hospital, Department Paediatric Surgery, London SW17 0QT, UK; kholmes@doctors.org.uk; 19 National Institute for Health Research University College London Hospitals Biomedical Research Centre, University College London Hospitals NHS Foundation Trust, Department of Oncology, London W1 2PG, UK; 20 IRCCS Istituto Giannina Gaslini, 16148 Genova, Italy; albertogaraventa@ospedale-gaslini.ge.it; 21 Institute of Cancer Research, Royal Marsden Hospital, Sutton SM5 2NG, UK; andy1pearson@btinternet.com; 22 Department of Pediatric Hematology and Oncology, University Medicine Greifswald, 17489 Greifswald, Germany; holger.lode@uni-greifswald.de

**Keywords:** high-risk neuroblastoma, immunotherapy, dinutuximab beta

## Abstract

To explore the effects of immunotherapy in the International Society of Paediatric Oncology Europe Neuroblastoma Group SIOPEN high-risk neuroblastoma 1 trial (HR-NBL1 trial), two cohorts were studied: one prior to and one after the introduction of dinutuximab beta. All patients received standard induction and high-dose therapy (HDT) with autologous stem cell rescue (ASCR); the local control comprised surgery and radiotherapy to the primary tumour site, followed by isotretinoin. A landmark timepoint of 109 days, resulting from the median time between ASCR and initiation of immunotherapy, was used to define patients’ eligibility in the pre-immunotherapy analysis cohort. Median follow-up was 5.8 years (inter-quartile range (IQR): 4.2–8.2 years) for 844 eligible patients balanced for risk factors, such as age, sex, stage 4, *MYCN* amplification and response prior to HDT. The five-year event-free and overall survival (95% confidence interval (CI) of 466 patients not receiving immunotherapy was 42% (38–47%) and 50% (46–55%) but was 57% (51–62%) and 64% (59–69%) for 378 patients receiving immunotherapy (*p* < 0.001). A multivariate analysis identified absence of immunotherapy (*p* = 0.0002, hazard ratio (HR) 1.573); type of HDT (*p* = 0.0029, HR 1.431); less than complete response prior to maintenance therapy (*p* = 0.0043, HR 1.494) and >1 metastatic compartment at diagnosis (*p* < 0.001, HR 2.665) as risk factors for relapse or progression. Results suggest an important role for dinutuximab beta-based immunotherapy within the treatment concepts applied in HR-NBL1/SIOPEN.

## 1. Introduction

High-risk neuroblastoma defined by metastatic diseases over the age of 12 or 18 months [1] and *MYCN* amplification at any age remain associated with long-term survival rates of only 40% [2,3]. Treatment approaches comprise intensive induction [4,5], consolidation with high-dose chemotherapy (HDT) and autologous stem cell rescue (ASCR) [3,6], and isotretinoin as maintenance therapy.

As the disialoganglioside GD_2_ is expressed on the majority of neuroblastoma cells, with minimal expression on normal cells, it is a suitable target for immunotherapy [7]. Therefore, human/mouse chimeric anti-GD_2_ antibody ch14.18, dinutuximab, produced in SP2/0 cells was developed and investigated in clinical trials [8]. In Europe, ch14.18 was re-cloned in Chinese hamster ovarian (CHO) cells (dinutuximab beta) [9] for clinical trials of International Society of Paediatric Oncology Europe Neuroblastoma Group SIOPEN. The tolerability and activity of dinutuximab beta was first evaluated in a dose schedule of 20 mg/m^2^ given on five consecutive days by an 8 h infusion [10]. In 2006, SIOPEN opened a randomised trial to compare dinutuximab beta and isotretinoin with isotretinoin alone in patients with high-risk neuroblastoma. However, in 2007, the results of the Children’s Oncology Group (COG) ANBL0032 trial were communicated, followed by publication in 2010 [7], demonstrating that two-year event-free survival (EFS) and overall survival (OS) of patients with high-risk neuroblastoma receiving dinutuximab and cytokines (granulocyte-macrophage colony stimulating factor and interleukin-2), in addition to isotretinoin, were significantly higher by 20% and 11%, respectively [7], compared to those patients receiving isotretinoin alone. Therefore, continuation of the SIOPEN randomised trial was believed to be no longer feasible nor considered ethical, and the study design was modified to allow all patients to receive dinutuximab beta with or without interleukin-2. The altered randomisation opened on 22 October 2009 to investigate the role of subcutaneous interleukin-2 (sc-IL-2) with dinutuximab beta and assigned patients to dinutuximab beta alone or with sc-IL-2 [11]. All patients received oral isotretinoin [12]. The trial showed that the addition of sc-IL-2 to immunotherapy with dinutuximab beta, given as an 8 h infusion, did not improve outcome but increased toxicity.

In this report, we aim to assess the contribution of dinutuximab beta-based immunotherapy to the outcome of patients with high-risk neuroblastoma in the International Society of Paediatric Oncology Europe Neuroblastoma Group High-Risk Neuroblastoma 1 (HR-NBL1/SIOPEN) trial by investigating the survival of patients in sequential eras with the same eligibility criteria treated with (immunotherapy population (IP), 2009–2013) [12] or without immunotherapy (control population (CP), 2002–2009).

## 2. Results

### 2.1. Patient Characteristics

**Figure 1:**
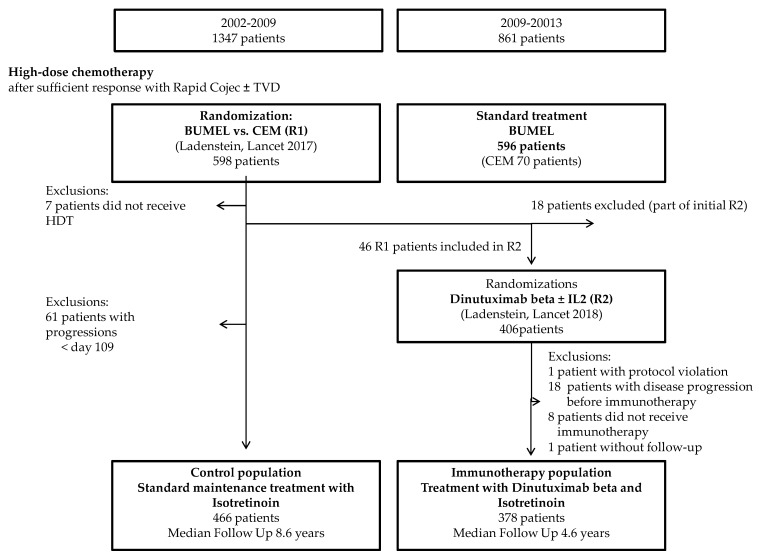
Flow chart for the analysis cohort. HDT (high-dose chemotherapy); BuMel (high-dose chemotherapy with busulfan and melphalan; CEM (high-dose chemotherapy with carboplatin, etoposide and melphalan); R1 (high-dose chemotherapy randomisation); R2 (immunotherapy randomisation) and IL-2 (interleukin-2).

According to the inclusion criteria for the analysis, 844 patients enrolled in 146 SIOPEN member hospitals/institutions in 19 countries were eligible (378 in the IP and 466 in the CP) (Figure 1). Median follow-up was 5.8 years (inter-quartile range (IQR): 4.2 to 8.2 years). The median age of patients at diagnosis was 2.9 years (IQR: 1.8. to 3.8).

Both populations were balanced for sex, stage 4, *MYCN* amplification and response prior to HDT (Table 1).

### 2.2. Survival

**Figure 2:**
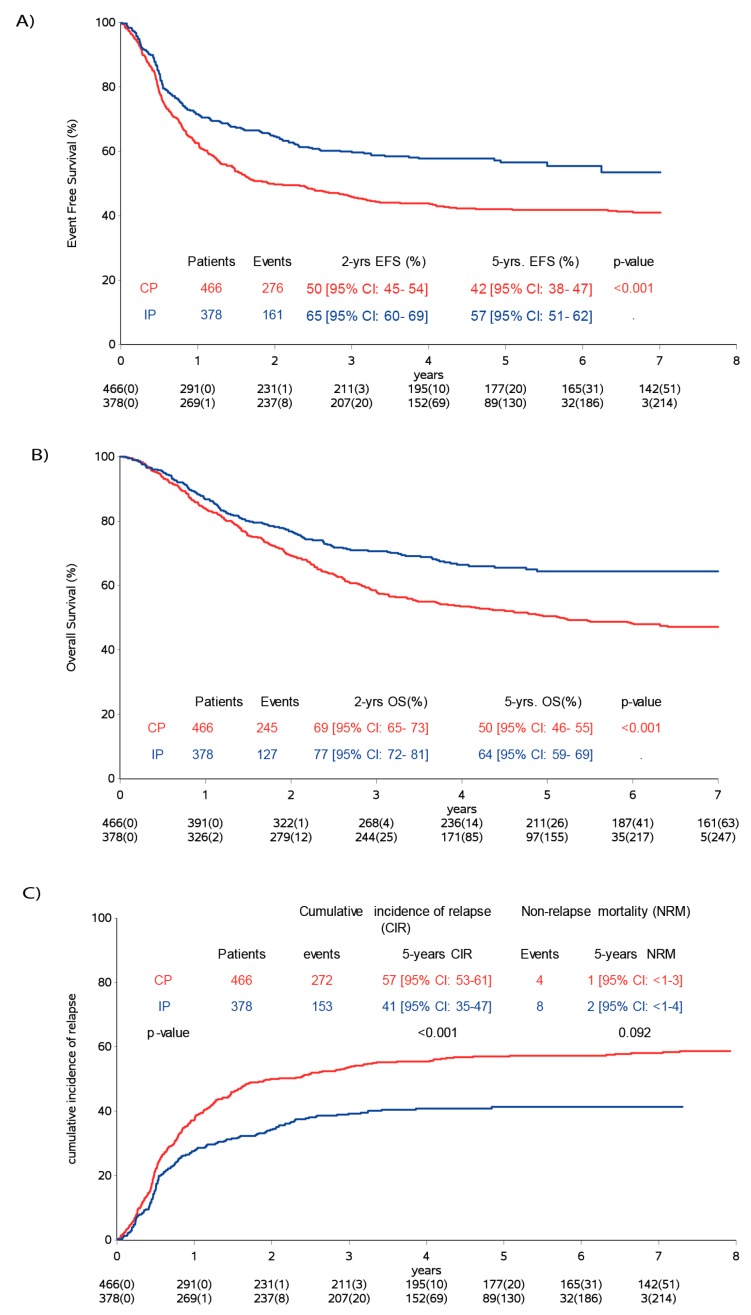
Analysis population comparing control population versus immunotherapy population. (**A**) Event-free survival, (**B**) overall survival and (**C**) cumulative incidence of progression/relapse. CP (control population), IP (immunotherapy population), CIR (cumulative incidence of relapse) and NRM (non-relapse mortality).

The five-year EFS was 57% (95% CI: 51–62%) for IP, compared to 42% (95% CI: 38–47%) for CP patients (*p* < 0.001) (Figure 2A). The five-year overall survival (OS) for the IP was 64% (95% CI: 59–69%), compared to 50% (95% CI: 46-55%) for CP patients (Figure 2B).

The cumulative incidence of relapse/progression (CIR) at five years was 41% (95% CI: 37–47%) for the IP and 57% (95% CI: 53–61%) for the CP patients (*p* < 0.001). At the last follow-up, 153 patients of the IP had an event versus 272 of CP patients. The cumulative incidence of non-relapse mortality was 2% (95% CI: 1–4%) in the IP and 1% (95% CI < 1–2%) in the CP (Figure 2C).

### 2.3. Influence of Risk Factors

**Figure 3:**
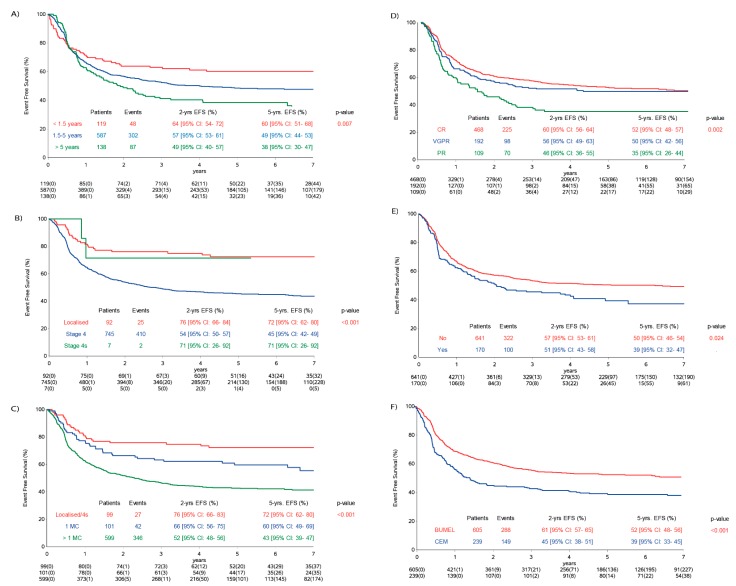
Influence of risk factors within the analysis population on event-free survival (EFS). (**A**) EFS and age; (**B**) EFS and stage; (**C**) EFS and metastatic compartments (MC) localised and 4s stage vs. stage 4 (one MC vs. multiple MC); (**D**) response status prior to maintenance phase: CR (complete remission), VGPR (very good partial remission) and PR (partial remission) and (**E**) EFS and TVD (topotecan, vincristine and doxorubicin). TVD added = yes and TVD not added = no. (**F**) Type of high-dose chemotherapy = BuMel (busulfan and melphalan) and CEM (carboplatin, etoposide and melphalan).

Disease status prior to maintenance therapy was available in 769/844 (91%) patients (Table 2). Older age; stage 4; involvement of more than one metastatic compartment (MC); disease status prior to maintenance therapy; addition of topotecan, vincristine and doxorubicin (TVD) and use of carboplatin, etoposide and melphalan (CEM) as HDT were associated with lower EFS (Figure 3) in the population analysed. Patients with lymph nodes as their only MC (five-year EFS 60% (95% CI: 36–78%)) had a similar EFS as patients with other isolated metastatic sites (five-year EFS 60% (95% CI: 49–70%)).

In the IP, the two-year and five-year EFS rates for 210 patients (81 events) in complete remission (CR) were 68% (95% CI: 61–74%) and 61% (95% CI: 53–57%). In the CP, the two-year and five-year EFS rates for 258 patients in CR (144 events) were 54% (95% CI: 48–60%) and 46% (95% CI: 39–52%).

The impact of immunotherapy on EFS was significantly influenced by stage and MC, and the impact of immunotherapy was stronger in patients with metastatic disease (Table 2). Furthermore, a borderline significant interaction between maintenance treatment and HDT (*p* = 0.055) was observed.

### 2.4. Multivariate Analysis on Analysis Cohort

Patients who had no immunotherapy (*p* = <.0001, cumulative hazard ratio (cHR) 1.75) HDT with CEM (*p* = 0.0345, cHR 1.3); partial remission (PR) prior maintenance therapy (*p* = 0.0103, cHR 1.49); more than one MC at diagnosis (*p* < 0.001, cHR 2.69) and age > 5 years (*p* = 0.0138, cHR 1.59) had a higher risk of relapse (Table 3).

After adjustment for age, stage, MC, TVD and response prior to maintenance therapy (Table 3), a benefit from immunotherapy was confirmed for either HDT (busulfan and melphalan (BuMel) or CEM). Patients receiving BuMel had an adjusted cumulative hazard ratio of 1.6 (1.2–2.1) with an unadjusted five-year EFS for the IP of 56% (95% CI: 50–61%) and 48% (95% CI: 41–54%) for the CP (*p* = 0.001). Patients receiving CEM had an adjusted cumulative hazard ratio of 3.0 (1.5–5.8) and showed an unadjusted five-year EFS of the IP of 67% (95% CI: 47–80%) versus 35% (95% CI: 29–42%) for the CP (*p* = 0.002).

### 2.5. Response to Maintenance Treatments

Thirty-nine of one hundred and eight (36%) CP patients with evaluable diseases prior to maintenance (67 very good partial remission (VGPR) and 41 partial remission (PR)) responded; of whom, 35/108 (32%) achieved CR after isotretinoin. In contrast, 64/130 (49%) of IP patients with evaluable diseases (85 VGPR and 45 PR) prior to immunotherapy responded; of whom, 52/130 (40%) achieved CR after immunotherapy (Table 4, *p* = 0.226).

### 2.6. Adverse Events and Toxicity

Adverse events (CTC Grades 1 to 4) are summarized in Table 5. Toxicity tended to be higher in the IP population, particularly in those patients who received sc-IL-2. Four patients had non-relapse-related mortality in the IP and four in the CP.

## 3. Discussion

This analysis showed superior EFS and OS in the era when dinutuximab beta-based immunotherapy was included in therapy for high-risk neuroblastoma, compared to the previous era in the same trial when isotretinoin alone was the only element of maintenance therapy. Although there are limitations with a historical comparison, this is the first and possibly only demonstration that the addition of dinutuximab beta as immunotherapy improves survival in the high-risk neuroblastoma front-line population treated homogenously in the HR-NBL1/SIOPEN trial. Both the control and immunotherapy populations received the same treatment approach. Furthermore, this analysis is an important contribution, as data on the efficacy of anti-GD_2_ antibody-based immunotherapy are limited to one prospective randomised trial and a few retrospective analyses. Ethical concerns precluded a randomised comparison of immunotherapy after the results of the COG ANBL0032 trial emerged. A randomised trial of dinutuximab beta and isotretinoin compared to isotretinoin alone would have produced more robust data, but this was believed not to be ethically feasible within the SIOPEN community.

As the two cohorts were from the same trial and using the same criteria; in particular, HDT within nine months from diagnosis and no progression at 109 days after ASCR as a starting point for survival, with an unchanged supportive care protocol guidance, this analysis provides important data supporting the benefit of dinutuximab beta-based immunotherapy. The landmark time identified was the median time observed between ASCR and initiation of dinutuximab beta; thus, only patients without progressive diseases at this timepoint were included in the pre-immunotherapy CP. The introduction of this landmark was important in order to exclude early relapse before immunotherapy could be commenced. Both populations, IP and CP, were balanced for age, sex, stage 4, *MYCN* amplification and response prior to HDT.

The benefit of immunotherapy was further underpinned by multivariate analysis. After adjustment for risk factors (for example, age, stage, MC at diagnosis, need for TVD and response prior to maintenance therapy) a positive impact on outcome was observed with either BuMel or CEM. This is particularly important in view of a higher percentage of patients receiving CEM in the control group, as we previously have shown superior outcomes for patients treated with BuMel [6]. Hence, the superior outcomes in the immunotherapy group are unlikely to be solely related to BuMel. These conclusions are supported by the recent publication from COG [13] demonstrating that immunotherapy improves survival even after optimised HDT regimens.

Response prior to immunotherapy is an important prognostic factor. Patients treated in CR in the IP had a two-year EFS of 68%, compared to 54% in the CP. Acknowledging that comparisons across trials are challenging, these results are similar to the previous report of dinutuximab in combination with IL-2 and GM-CSF in patients in CR, resulting in a two-year EFS of 66% for patients treated by immunotherapy, compared to only 46% in patients with an isotretinoin maintenance treatment [7]. Further support in favour of dinutuximab beta-based immunotherapy within the HR-NBl1/SIOPEN trial comes from improved response rates in patients with residual diseases at the site of the primary tumour or metaiodobenzylguanidine (mIBG)-positive skeletal disease following immunotherapy; a 49% response rate and a 40% CR rate was observed in patients treated with immunotherapy, as compared to a 36% overall response and 32% CR rate in the control population.

The increasing role for immunotherapy, with anti-GD_2_ antibodies, in the therapy of patients with high-risk neuroblastoma is further highlighted by the recent demonstration of the efficacy of combining anti-GD_2_ antibodies with chemotherapy, either at relapse or at initial presentation [14,15].

In summary, this report describes the effects of including dinutuximab beta in high-risk neuroblastoma maintenance therapy and shows a clear survival benefit. This provides an important baseline to further build immunotherapy strategies in this challenging patient population.

## 4. Materials and Methods

### 4.1. Trial Eligibility

HR-NBL1/SIOPEN, an international, randomised, multiarm, open-label, phase 3 trial for high–risk neuroblastoma, opened on 24 June 2002 and is registered with ClinTrials.gov, number NCT01704716, and EudraCT, number 2006-001489-17. All randomisations of the HR-NBL1/SIOPEN trial are closed, and three have been published [5,6,12]. SIOPEN institutions recruited patients after approval of the trial by national regulatory authorities and ethical committees. Parents/guardians and patients provided written informed consent or assent, when applicable.

The International Neuroblastoma Staging System criteria (INSS) and International Neuroblastoma Response Criteria (INRC) [16] were used to classify the disease and to evaluate responses to therapy. Untreated patients with INSS stage 4 metastatic neuroblastoma aged 1-20 years or INSS stage 2–4 neuroblastoma with *MYCN* amplification, as determined in SIOPEN reference laboratories [17], any age up to 20 years, were eligible. The SIOPEN-R-NET web-based system (https://www.siopen-r-net.org/) randomly assigned eligible patients in real-time.

### 4.2. Eligibiliy for the Analysis Cohort and Treatments Given

This analysis included all patients registered in the HR-NBL1/SIOPEN trial between 2002 and 2013 who met the criteria: (i) HDT within 9 months from diagnosis and (ii) no progression at 109 days after ASCR. The median time between ASCR and initiation of dinutuximab beta was 109 days; therefore, only patients without progressive diseases at this landmark timepoint were included in the pre-immunotherapy control population (CP). The 18 patients randomised to receive dinutuximab beta and isotretinoin between 2006 and 2009 were excluded from this analysis. Two cohorts were compared, a CP between 2002–2009 who did not receive dinutuximab beta and an IP between 2009 and 2013 who was randomised to receive dinutuximab beta with or without sc-IL2 [12]. As the addition of IL-2 to immunotherapy with dinutuximab beta did not improve outcome, both randomised arms were considered as one group for the purposes of this analysis [12]. The CP comprised patients who received HDT randomised to BuMel or CEM [6] but did not receive immunotherapy.

**Figure 4:**
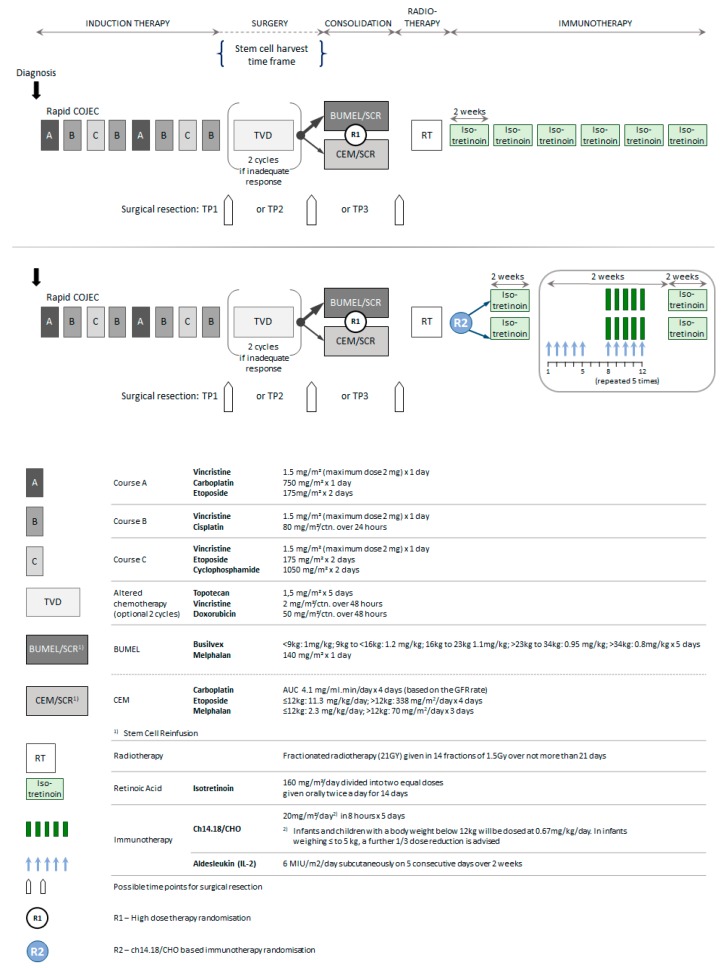
HR-NBL1/SIOPEN treatment overview.

All patients received rapid COJEC induction [4,5] (Figure 4). Between 2002 to 2010, patients who had a bone marrow complete response (CR) and a metastatic CR or a metastatic partial response (PR) defined by INSS criteria but at least 50% reduction in skeletal metaiodobenzylguanidine (mIBG) positivity from baseline and three or fewer areas of abnormal uptake on ¹²³I-mIBG scintigraphy were eligible for HDT randomisation comparing BuMel with CEM [6]. Thereafter, BuMel became the standard of care. Patients who did not achieve a metastatic response to fulfil the HDT eligibility criteria received two courses of topotecan, vincristine and doxorubicin (TVD) [18] prior to HDT. Local treatment of the primary tumour comprised attempted total surgical resection and radiotherapy (21 Gy) to the primary tumour site between 60 and 90 days after ASCR. There was no dose modification in the event of incomplete tumour excision; neither were metastatic sites systematically irradiated. 

All patients received 6 cycles of oral isotretinoin over two weeks [3] after local irradiation. From 2009, patients were randomised between day 60 to 90 after ASCR to receive five courses of dinutuximab beta at a dose of 20 mg/m^2^/day as an 8 h infusion for 5 consecutive days (total dose of 100 mg/m² per cycle, days 8 to 12) with or without 6 × 10^6^IU/m^2^/day of sc-IL-2 on days 1 to 5 and days 8 to 12 of each immunotherapy cycle [12].

Patients had full disease evaluations prior to and after 2 and 5 courses of maintenance treatment. This included whole body ^123^I-mIBG scintigraphy, CT or MRI scans of the primary tumour and any other evaluable site of the disease; bone marrow examination with both aspirates and trephines obtained from two sites and measurement of urinary catecholamine metabolites. Response was assessed by the 1993 INRC based on local institution reporting. The only evaluable diseases prior to immunotherapy were mIBG-positive skeletal disease or diseases detectable on CT/MRI scans prior to randomisation, as patients with bone marrow involvement were not eligible.

### 4.3. Statistical Analysis 

#### 4.3.1. Establishment of the Analysis Cohort

The number of MC at diagnosis was calculated according to the number of MC at diagnosis either in the bone marrow, skeleton or other sites, with a possible range from one to six. Characteristics of the CP were compared to the IP by the chi-square test to assess the balance of risk factors between the two populations.

#### 4.3.2. Outcome Parameters

Follow-up commenced at 109 days for the CP cohort and from the first dose of dinutuximab beta for the IP cohort. EFS and OS were estimated using the Kaplan and Meier method and compared with the log-rank test [19]. CIR was estimated [20], taking into account the competing risk of death without relapse/progression. For cumulative incidence of non-relapse mortality, relapse or progression was considered as a competing event. The statistical comparison of cumulative incidences used Gray’s methodology [21]. EFS, OS, cumulative incidence of relapse/progression and cumulative incidence of non-relapse mortality are presented as 5-year point estimates with confidence intervals (CI), as previously described [20,22]. Two-year EFS was determined to facilitate comparisons with published data [7]. *p*-values of less than 0.05 were considered to indicate statistical significance. In spite of the limitations of subgroup analysis (including multiple testing and lack of power), the cohorts were assessed according to baseline, pre and post-HDT risk factors (Table 2). A formal test for interaction was performed within a Cox model.

#### 4.3.3. Multivariate Analysis

Multivariate analysis of treatment and risk factors was undertaken on the analysis cohort. In the presence of nonproportional hazards, as detected for age and *MYCN* amplification, the pseudo-value regression [22] for a 5-year EFS approach was chosen. The aim of this analysis was to adjust the comparison between the two populations (IP and CP) for potential confounders and risk factors, such as age, stage, addition of TVD, disease status prior to maintenance and HDT/ASCR. Using the same approach, a subgroup analysis was performed in order to separately evaluate the value of immunotherapy in patients with BuMel and CEM. 

The data cut-off time of this analysis was July 31, 2017. Median follow-up was calculated using the inverse Kaplan Meier estimate. The statistical evaluation and power calculation were done with SAS 9.4 and Module LR1 of Pass 2002, respectively.

## 5. Conclusions

This report shows that the introduction of dinutuximab beta is associated with a survival benefit for children with high-risk neuroblastoma. Similar results were reported for dinutuximab in one randomized trial and one nonrandomized investigation [23,24,25]. However, dinutuximab beta is a different molecule with a separate development pathway, and the demonstration of its beneficial effects on treatment outcome is an important finding.

Given the absence of a beneficial effect by adding sc-IL-2 to an 8 h infusion of dinutuximab beta [12], the standard treatment recommended by SIOPEN is dinutuximab beta with isotretinoin for maintenance therapy of high-risk neuroblastoma. The benefits might be less in some subgroups (<1.5 years, *MYCN*-amplified localised disease) and needs close monitoring in future studies. Modifications of the length of the dinutuximab beta schedule and immunotherapy combination strategies may optimise the benefits of immunotherapy in high-risk neuroblastoma to improve survival.

## Figures and Tables

**Table 1:** Characteristics of the control and immunotherapy populations. N = number; % = percentage; MNA = *MYCN* amplification; no = not present and yes = present; MC = metastatic compartments; TVD = topotecan, vincristine and doxorubicin; HDT = high-dose chemotherapy; BuMel = high-dose chemotherapy with busulfan and melphalan; CEM = high-dose chemotherapy with carboplatin, etoposide and melphalan; NR = not reported; CR = complete remission; VGPR = very good partial remission; PR = partial remission; CME = complete macroscopic excision and IME = incomplete macroscopic excision.

Characteristics		Control Population		Immunotherapy Population	
		*n*	%	*n*	%
**Total**	number	466		378	
**Sex**	Female	180	39%	140	37%
	Male	286	61%	238	63%
**Age**	<1.5 years	64	13%	55	14%
	1.5-<5 years	333	71%	254	67%
	≥5 years	69	15%	69	18%
	Median	2.70		2.87	
**Stage**	Localised	60	13%	32	8%
	Stage 4	406	87%	339	90%
	Stage 4s	0	0%	7	2%
***MYCN Stage 4***	*MNA NR*	27	6%	16	4%
	MNA no	217	57%	197	61%
	MNA yes	162	43%	126	39%
**MC**	NR	23	5%	23	6%
	0	60	14%	32	9%
	1	70	16%	35	10%
	2	136	31%	112	32%
	3	120	27%	112	32%
	>3	57	13%	64	18%
**TVD given**	NR	23	5%	10	3%
	No	391	88%	250	68%
	Yes	52	12%	118	32%
**Surgery**	CME	318	76%	261	75%
	IME	101	24%	87	25%
**Status prior HDT**	NR	25	5%	33	9%
	CR	174	39%	116	34%
	VGPR	159	36%	149	43%
	PR	108	24%	80	23%
**HDT**	BuMel	257	55%	348	92%
	CEM	209	45%	30	8%
**Status prior Maintenance**	NR	58	12%	17	4%
	CR	258	63%	210	58%
	VGPR	93	23%	99	27%
	PR	57	14%	52	14%

**Table 2:** Outcomes according to risk factors and subgroups. (2A) Event-free survival and (2B) overall survival. Pts (patients); 95% CI (95% confidence interval); *p*- value (probability value for A: comparison according to risk factor and B: for interaction); MNA (*MYCN* amplification); - (not present) and + (present); MC (metastatic compartments); TVD (topotecan, vincristine and doxorubicin); HDT (high-dose chemotherapy); BuMel (high-dose chemotherapy with busulfan and melphalan); CEM (high-dose chemotherapy with carboplatin, etoposide and melphalan); CR (complete remission); VGPR (very good partial remission; PR (partial remission); CME (complete macroscopic excision) and IME (incomplete macroscopic excision).

Characteristics			Total Population			Control Group		Immunotherapy Group		*p*-Value ^B^
(A) Event Free Survival			Events/Pts	5-years EFS (95% CI)	*p*-value ^A^	Events/Pts	5-years EFS (95% CI)	Events/Pts	5-years EFS (95% CI)	
**Total**			**844 Pts**			**466 Pts**		**378 Pts**		
**Sex**	female		164/320	49 (44–55)	0.803	105/180	43 (36–50)	59/140	57 (49–65)	0.938
	male		273/524	48 (44–52)		171/286	41 (36–47)	102/238	56 (49–62)	
**Age**	<1.5 years		48/119	60 (51–68)	0.007	27/64	59 (46–70)	21/55	61 (47–73)	0.161
	1.5–<5 years		302/587	49 (44–53)		194/333	42 (37–47)	108/254	57 (50–63)	
	≥5 years		87/138	38 (30–47)		55/69	25 (16–36)	32/69	53 (40–64)	
**Stage**	Localised		25/92	72 (62–80)	<0.001	14/60	76 (63–85)	11/32	66 (47–79)	0.007
	Stage 4		410/745	45 (42–49)		262/406	37 (32–42)	148/339	56 (50–61)	
	Stage 4s		2/7	71 (26–92)		–	–	2/7	71 (26–92)	
	Stage 4 MNA no–		236/414	43 (38–48)	0.819	147/217	33 (27–39)	89/197	54 (47–61)	0.25
	Stage 4 MNA yes+		154/288	47 (41–53)		98/162	42 (34–49)	56/126	55 (45–63)	
**MC**	0		25/92	72 (62–80)	<0.001	14/60	67 (63–85)	11/32	66 (47–79)	0.025
	1		43/105	60 (50–69)		34/70	54 (42–65)	9/35	71 (50–85)	
	2		136/248	46 (39–52)		85/136	39 (31–47)	51/112	55 (45–63)	
	3		134/232	43 (36–49)		89/120	27 (20–36)	45/112	60 (50–69)	
	>3		76/121	37 (28–46)		42/57	28 (17–40)	34/64	45 (32–57)	
**TVD**	no		322/641	50 (46–54)	0.024	225/391	44 (39–49)	97/250	61 (54–67)	0.732
	yes		100/170	39 (32–47)		38/52	27 (16–39)	62/118	45 (34–55)	
**Surgery**	CME		288/578	50 (46–54)	0.123	183/318	43 (38–49)	105/260	59 (52–65)	0.946
	IME		106/188	45 (38–52)		66/101	38 (29–48)	40/87	53 (42–63)	
**Status Prior HDT**										
	CR		134/290	54 (48–59)	0.022	86/174	51 (43–58)	48/116	57 (47–66)	0.294
	VGPR		171/308	45 (39–51)		104/159	36 (29–44)	67/149	55 (46–63)	
	PR		104/188	45 (38–53)		70/108	38 (29–47)	34/80	57 (45–67)	
**Characteristics**			**Total Population**			**Control Group**		**Immunotherapy Group**		***p*** **–value ^B^**
**(A) Event Free Survival**			**Events/Pts**	**5–years EFS (95% CI)**	***p*** **–value ^A^**	**Events/Pts**	**5–years EFS (95% CI)**	**Events/Pts**	**5–years EFS (95% CI)**	
**HDT**	BuMel		288/605	52 (48–56)	<0.001	137/257	48 (41–54)	151/348	56 (50–61)	0.055
	CEM		149/239	39 (33–45)		139/209	35 (29–42)	10/30	67 (47–80)	
**Status Prior Maintenance**										
	CR		225/468	52 (48–47)	0.002	144/258	46 (39–52)	81/210	61 (53–67)	0.84
	VGPR		98/192	50 (42–56)		55/93	43 (33–53)	43/99	56 (46–66)	
	PR		70/109	35 (26–44)		42/57	26 (15–33)	28/52	45 (32–58)	
**(B) Overall Survival**			**Events/ Pts**	**5-year OS (95% CI)**	***p*** **-value ^A^**	**Events/Pts**	**5-year OS (95% CI)**	**Events/Pts**	**5-year OS (95% CI)**	
**Sex**		female	138/320	59 (53–64)	0.536	95/180	53 (45–60)	43/140	59 (53–64)	0.488
		male	234/524	55 (50–59)		150/286	49 (43–55)	84/238	55 (50–59)	
**Age**		<1.5 years	45/119	62 (52–70)	0.445	26/64	61 (48–72)	19/55	62 (52–70)	0.409
		1.5–<5 years	259/587	57 (53–61)		177/333	50 (45–55)	82/254	57 (53–61)	
		≥5 years	68/138	49 (40–58)		42/69	42 (30–54)	26/69	49 (40–58)	
**Stage**		localised	24/92	76 (66–83)	0.001	13/60	82 (69–89)	11/32	76 (66–83)	0.003
		Stage 4	347/745	54 (50–57)		232/406	46 (41–51)	115/339	54 (50–57)	
		Stage 4s	0/7	83 (27–97)		0/0		1/7		
		Stage 4 MNA no-	193/414	53 (47–58)	0.235	129/217	43 (37–50)	64/197	53 (47–58)	0.150
		Stage 4 MNA yes+	137/288	54 (48–60)		88/162	49 (41–56)	49/126	54 (48–60)	
**MC**		0	24/92	76 (66–83)	<0.001	13/60	82 (69–89)	11/32	76 (66–83)	0.013
		1	36/105	68 (58–76)		29/70	62 (50–73)	7/35	68 (58–76)	
		2	109/248	56 (50–62)		71/136	51 (42–59)	38/112	56 (50–62)	
		3	117/232	49 (42–56)		81/120	36 (27–44)	36/112	49 (42–56)	
		>3	70/121	43 (34–52)		41/57	32 (20–44)	29/64	43 (34–52)	
**TVD**		no	281/641	57 (53–61)	0.224	200/391	52 (47–57)	81/250	57 (53–61)	0.441
		yes	78/170	52 (44–60)		34/52	36 (23–49)	44/118	52 (44–60)	
**Surgery**		CME	248/578	57 (53–61)	0.218	165/318	51 (45–56)	83/260	57 (53–61)	0.765
		IME	91/188	52 (45–59)		58/101	46 (36–55)	33/87	52 (45–59)	
**Status Prior HDT**										
		CR	116/290	60 (54–66)	0.089	80/174	56 (48–63)	36/116	60 (54–66)	0.522
		VGPR	140/308	55 (49–61)		86/159	50 (42–58)	54/149	55 (49–61)	
		PR	92/188	52 (45–59)		65/108	44 (34–53)	27/80	52 (45–59)	
**HDT**		BUMEL	238/605	60 (56–64)	<0.001	121/257	56 (50–62)	117/348	60 (56–64)	0.267
		CEM	134/239	46 (40–53)		124/209	44 (37–50)	10/30	46 (40–53)	
**Characteristics**			**Total Population**			**Control Group**		**Immunotherapy Group**		***p*** **–value ^B^**
**(B) Overall Survival**			**Events/ Pts**	**5-year OS (95% CI)**	***p*** **-value ^A^**	**Events/Pts**	**5-year OS (95% CI)**	**Events/Pts**	**5-year OS (95% CI)**	
**Status Prior Maintenance**										
		CR	190/468	60 (55–65)	0.006	129/258	54 (47–59)	61/210	60 (55–65)	0.640
		VGPR	83/192	57 (49–64)		47/93	52 (42–62)	36/99	57 (49–64)	
		PR	61/109	43 (33–53)		38/57	36 (23–48)	23/52	50 (33–65)	

**Table 3:** Multivariate analysis of the analysis cohort. cHR (cumulative hazard ratios); 95% CI (95% confidence interval); *p*-value (probability value); MNA (*MYCN* amplification); MC (metastatic compartments, referring either to bone marrow, skeletal or lymph node involvement); TVD (topotecan, vincristine and doxorubicin); CR (complete remission); VGPR (very good partial remission); PR (partial remission); HDT (high-dose chemotherapy); BuMel (high-dose chemotherapy with busulfan and melphalan); CEM (high-dose chemotherapy with carboplatin, etoposide and melphalan); IP (immunotherapy population) and CP (control population). * test for the global main effect for risk-factors with more than two categories.

	Risk Factor	Characteristics	Pseudo Values for 5-Years EFS	
			cHR (95% CI)	*p*-Value
**(A)**	**Multivariate Analysis**			
	**Immunotherapy vs. Control Cohort**		1.75 (1.36–2.25)	<0.0001
	**Age (vs. <1.5 yrs)**			0.0931 *
		**1.5–5 years**	1.31 (0.92–1.87)	0.1384
		**>5years**	1.59 (1.05–2.42)	0.0138
	**Stage 4, 4s and Number of MC (vs. MNA stages 2, 3)**			<0.0001 *
		**1 MC**	1.38 (0.80–2.47)	0.2493
		**>1MC**	2.69 (1.74–4.15)	<0.0001
	**TVD**		1.28 (0.97–1.69)	0.2478
	**Status Prior Maintenance (vs. CR)**			0.0363 *
		**VGPR**	1.06 (0.81–1.39)	0.6416
		**PR**	1.49 (1.10–2.02)	0.0103
	**HDT**	**CEM vs. BuMel**	1.32 (1.02–1.70)	0.0345
**(B)**	**Subgroup Analysis According to HDT(after adjustment for age, stage, MC, TVD and status prior maintenance treatment)**			
	**BUMEL**	**IP vs. CP**	1.6 (1.2–2.1)	0.001
	**CEM**	**IP vs. CP**	3.0 (1.5–5.8)	0.002

**Table 4:** Response for immunotherapy and control populations. Legend: CR (complete remission), VGPR (very good partial remission), PR (partial remission), SD (stable disease) and PD (progressing disease).

Response Status before Maintenance			Response Status after Maintenance					
		Total	Evaluable	CR	VGPR	PR	SD	PD
				**Immunotherapy and Isotretinoin**				
**CR**		**210**	**188**	**151**	**0**	**0**	**0**	**37**
**<CR**	**Total**	**151**	**130**	**52**	**43**	**8**	**0**	**27**
	**VGPR**	99	85	36	31	0	0	18
	**PR**	52	45	16	12	8	0	9
				**Isotretinoin**				
**CR**		**258**	204	163	0	0	0	40
**<CR**	**Total**	**150**	**108**	**35**	**27**	**18**	**1**	**27**
	**VGPR**	93	67	28	23	2	0	14
	**PR**	57	41	7	4	16	1	13

**Table 5:** Toxicities IL-2 (interleukin 2), non-hem. tox. (non-hematological toxicities), WBC (white blood cells), ECHO LV (left ventricular)/SV (stroke volume), GFR (glomerular filtration rate), central neuro (central neurotoxicity), periph neuro (peripheral neurotoxicity) and liver enzymes: SGOT (serum-glutamat-oxalacetat-transaminase)/SGPT (serum-glutamat-pyruvat-transaminase). Columns in bold show the values for the combined grade 3 and 4 toxicities.

Toxicities	Control								Without IL2								With IL2							
	Eval								All								All							
		0	1	2	3	4	3 + 4			0	1	2	3	4	3 + 4			0	1	2	3	4	3 + 4	
**Non-Hem. Tox.**	317	113	42	116	41	5	**46**	**15%**	186	5	6	53	105	17	**122**	**66%**	192	4	2	20	113	53	**166**	**86%**
**General Condition**	314	225	69	13	4	3	**7**	**2%**	185	42	70	43	24	6	**30**	**16%**	192	25	53	36	66	12	**78**	**41%**
**Haemoglobin**	313	208	39	54	8	4	**12**	**4%**	186	21	2	84	69	10	**79**	**42%**	191	14	5	46	102	24	**126**	**66%**
**WBC**	313	235	32	28	15	3	**18**	**6%**	186	35	30	73	42	6	**48**	**26%**	191	35	30	57	51	18	**69**	**36%**
**Granulocytes**	313	244	23	25	16	5	**21**	**7%**	186	44	26	54	43	19	**62**	**33%**	191	34	12	34	69	42	**111**	**58%**
**Platelets**	313	260	13	12	16	12	**28**	**9%**	186	66	25	31	40	24	**64**	**34%**	191	30	18	26	61	56	**117**	**61%**
**Infection**	315	220	52	23	19	1	**20**	**6%**	185	79	26	32	47	1	**48**	**26%**	191	60	20	47	58	6	**64**	**34%**
**Fever**	314	241	14	54	4	1	**5**	**2%**	185	41	3	116	24	1	**25**	**14%**	190	28	4	82	66	10	**76**	**40%**
**Stomatitis**	312	293	10	7	2	0	**2**	**1%**	185	156	18	8	0	3	**3**	**2%**	191	149	27	12	2	1	**3**	**2%**
**Nausea/Vomiting**	313	284	6	19	4	0	**4**	**1%**	185	88	11	76	9	1	**10**	**5%**	191	68	12	94	14	3	**17**	**9%**
**Diarrhoea**	313	286	11	13	3	0	**3**	**1%**	185	93	32	47	10	3	**13**	**7%**	192	75	25	51	34	7	**41**	**21%**
**Constipation**	312	302	7	3	0	0	**0**	**0%**	185	110	43	32	0	0	**0**	**0%**	191	142	21	21	4	3	**7**	**4%**
**Skin**	315	181	50	73	10	1	**11**	**3%**	185	65	46	65	9	0	**9**	**5%**	192	48	48	77	19	0	**19**	**10%**
**Allergy**	314	307	4	3	0	0	**0**	**0%**	185	88	50	28	14	5	**19**	**10%**	191	75	39	38	32	7	**39**	**20%**
**Cardiac Function**	298	298	0	0	0	0	**0**	**0%**	182	178	0	0	3	1	**4**	**2%**	191	183	3	1	3	1	**4**	**2%**
**Echo LV/SV**	298	298	0	0	0	0	**0**	**0%**	182	181	0	0	0	1	**1**	**1%**	189	181	5	2	0	1	**1**	**1%**
**Hypotension**	298	296	2	0	0	0	**0**	**0%**	182	139	22	8	12	1	**13**	**7%**	191	119	23	17	25	7	**32**	**17%**
**Hypertension**	298	298	0	0	0	0	**0**	**0%**	182	162	10	3	7	0	**7**	**4%**	190	177	4	6	3	0	**3**	**2%**
**Creatinine**	312	301	9	2	0	0	**0**	**0%**	185	167	14	1	3	0	**3**	**2%**	192	159	20	11	2	0	**2**	**1%**
**Proteinuria**	311	307	4	0	0	0	**0**	**0%**	184	169	13	2	0	0	**0**	**0%**	191	178	12	1	0	0	**0**	**0%**
**Haematuria**	311	305	6	0	0	0	**0**	**0%**	183	167	11	5	0	0	**0**	**0%**	191	169	16	6	0	0	**0**	**0%**
**GFR**	310	302	5	3	0	0	**0**	**0%**	183	172	6	2	3	0	**3**	**2%**	190	179	9	1	1	0	**1**	**1%**
**Central Neuro**	311	304	4	0	0	3	**3**	**1%**	185	165	14	3	3	0	**3**	**2%**	191	158	16	6	3	8	**11**	**6%**
**Periph Neuro**	311	308	1	0	1	1	**2**	**1%**	185	173	8	3	1	0	**1**	**1%**	191	167	14	4	5	1	**6**	**3%**
**Bilirubin**	309	301	4	2	2	0	**2**	**1%**	185	169	1	10	4	1	**5**	**3%**	192	159	5	21	6	1	**7**	**4%**
**SGOT/SGPT**	311	218	68	19	6	0	**6**	**2%**	185	68	43	43	30	1	**31**	**17%**	192	68	40	40	43	1	**44**	**23%**
**Dilated Pupils**	22	22	0	0	0	0	**0**	**0%**	123	108	15	0	0	0	**0**	**0%**	125	95	30	0	0	0	**0**	**0%**
**Accommodation Defects**	22	22	0	0	0	0	**0**	**0%**	121	115	6	0	0	0	**0**	**0%**	125	111	14	0	0	0	**0**	**0%**
**Capillary Leak Syndrome**	19	18	0	1	0	0	**0**	**0%**	119	91	0	23	5	0	**5**	**4%**	124	70	0	35	16	3	**19**	**15%**
**Cytokine Release Syndrome**	19	18	1	0	0	0	**0**	**0%**	118	95	8	10	5	0	**5**	**4%**	123	85	12	17	9	0	**9**	**7%**
**Pain related to ch14.18/CHO**									122	42	17	44	19	0	**19**	**16%**	124	28	22	42	31	1	**32**	**26%**
**Papilloedema**	22	22	0	0	0	0	**0**	**0%**	120	113	7	0	0	0	**0**	**0%**	123	121	2	0	0	0	**0**	**0%**
